# Mitochondrial Dysfunction in Spinal Muscular Atrophy

**DOI:** 10.3390/ijms231810878

**Published:** 2022-09-17

**Authors:** Eleonora Zilio, Valentina Piano, Brunhilde Wirth

**Affiliations:** 1 Institute of Human Genetics, University Hospital of Cologne, University of Cologne, 50931 Cologne, Germany; 2 Center for Molecular Medicine Cologne, University of Cologne, 50931 Cologne, Germany; 3 Institute for Genetics, University of Cologne, 50674 Cologne, Germany; 4 Center for Rare Diseases, University Hospital of Cologne, University of Cologne, 50931 Cologne, Germany

**Keywords:** mitochondria, spinal muscular atrophy, *SMN1*, *SMN2*, motor neuron diseases, neurodegeneration, cellular homeostasis, neurodegenerative diseases, oxidative stress, mitochondria biogenesis and dynamics

## Abstract

Spinal muscular atrophy (SMA) is a devastating neuromuscular disorder caused by recessive mutations in the *SMN1* gene, globally affecting ~8–14 newborns per 100,000. The severity of the disease depends on the residual levels of functional survival of motor neuron protein, SMN. SMN is a ubiquitously expressed RNA binding protein involved in a plethora of cellular processes. In this review, we discuss the effects of SMN loss on mitochondrial functions in the neuronal and muscular systems that are the most affected in patients with spinal muscular atrophy. Our aim is to highlight how mitochondrial defects may contribute to disease progression and how restoring mitochondrial functionality may be a promising approach to develop new therapies. We also collected from previous studies a list of transcripts encoding mitochondrial proteins affected in various SMA models. Moreover, we speculate that in adulthood, when motor neurons require only very low SMN levels, the natural deterioration of mitochondria associated with aging may be a crucial triggering factor for adult spinal muscular atrophy, and this requires particular attention for therapeutic strategies.

## 1. Introduction

Spinal muscular atrophy (SMA) is a genetically common autosomal recessively inherited motor neuron (MN) disease, which in its natural development causes early childhood lethality in approximately half of all SMA patients [1]. It affects the proximal voluntary muscles symmetrically and extends to the distal muscles as the disease progresses [2,3]. There is increasing evidence of multisystem involvement, particularly in its severe form of manifestation [4]. The age-of-onset of SMA is very broad, ranging from prenatal (type 0) to the first 6 months of life (type I), after 6 months of life (type II), after 18 months of life (type III) or after 25 years (type IV). Motoric abilities range from never able to sit or stand (type 0 and I), able to sit (type II) and able to walk (type III and IV) [2,3].

**Figure 1:**
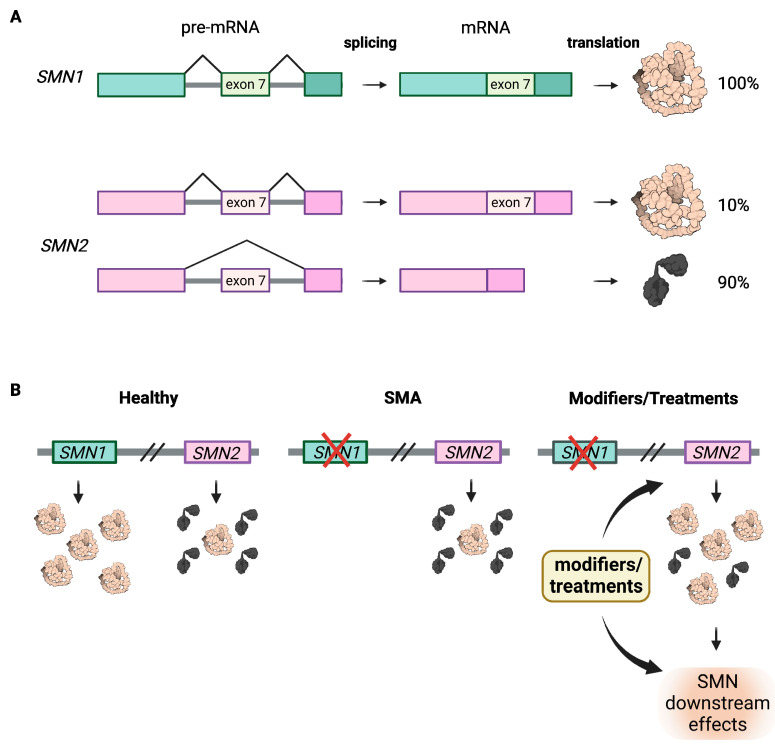
Genetic basis of SMA. (**A**) *SMN1* and *SMN2* genes are located on chromosome 5q13 and encode for SMN protein. In healthy conditions, the *SMN1* pre-mRNA undergoes correct splicing, while *SMN2* pre-mRNA mainly undergoes erroneous splicing, which results in the loss of the exon 7 and the translation of a truncated and non-functional SMN protein. (**B**) Healthy individual present physiological levels of SMN protein, deriving mainly from the *SMN1* gene. In SMA patients, deletion of or missense mutations in *SMN1* cause a dramatic reduction in SMN protein level that cannot be compensated by the *SMN2* copy gene. The SMN protein levels can be (partially) restored by genetic modifiers or treatments that either boost the production of SMN protein (*SMN2* copy number is a modifier in this sense) or act on the downstream functions of SMN to compensate its loss. The figure was created with BioReneder.com.

SMA is caused by homozygous loss of function of *SMN1* [5], while disease severity is mainly determined by *SMN2* copy number; both *SMN* genes are located close together on chromosome 5q13 [6,7]. *SMN2* is an almost perfect copy of *SMN1* with five nucleotide differences, only one of which is relevant as it affects splicing by disrupting an exonic splicing enhancer and creating a new exonic splicing silencer in exon 7 [8,9,10]. Consequently, 90% of *SMN2* transcripts lack exon 7 and produce a protein that is truncated and unstable [11]. About 10% of *SMN2* transcripts are correctly spliced and translated into a full-length protein identical to that produced by *SMN1* (Figure 1A). However, the low SMN protein amount in patients cannot counteract the loss of *SMN1*, thus primarily affecting spinal motor neurons, which require high levels of SMN prenatally and in the first three months of life but very little in adolescence and adulthood [1,12].

Although all SMA patients have a homozygous loss of functional *SMN1*, the copy number of *SMN2* varies between 1–6, which is inversely correlated with disease severity [6,7]. The more *SMN2* copies a patient has, the milder the phenotype. Therefore, *SMN2* is the main modulator of the SMA disease phenotype and the main target for therapies (Figure 1B).

To date, three FDA- and EMA-approved drugs have been shown to be effective in ameliorating or restoring motor abilities, particularly if the therapy begins pre-symptomatically, as is the case when newborn screening has been implemented [13,14,15]. All three therapies aim to increase SMN levels, either through (i) antisense oligonucleotides that correct splicing by blocking an intronic silencer in intron 7 (intrathecal injection of nusinersen/Spinraza, Ionis Pharmaceuticals and Biogen), (ii) small molecules facilitating the recruitment of U1-snRNPs to the splice donor of intron 7 (systemic oral application of risdiplam/Evrisdy, Gentechen and Roche), or (iii) gene replacement therapy, using a self-complementary AAV9 expressing *SMN1* cDNA under a strong ubiquitous promotor (systemic intravenous application of onasemnogene abeparvovec xioi/Zolgensma, AveXis and Novartis) [16,17,18,19,20,21].

**Figure 2:**
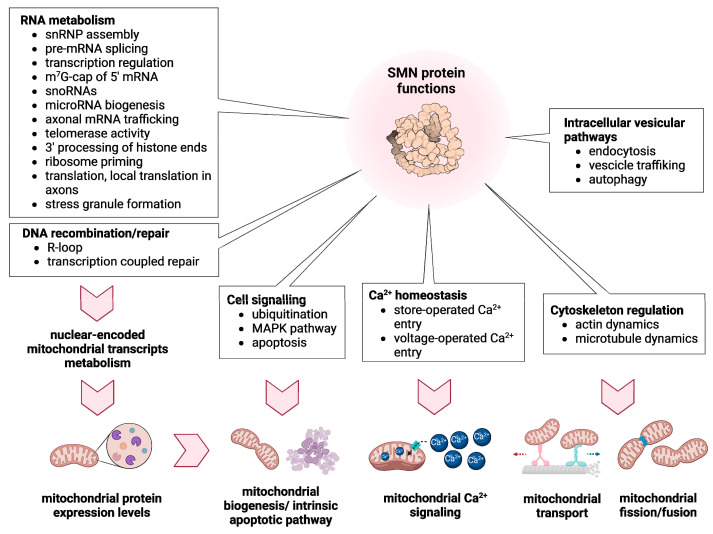
Schematic summary of the known functions of SMN and their impact on mitochondrial homeostasis. As crucial regulator of RNA metabolism and DNA recombination/repair, SMN modulates the expression levels of many mitochondrial proteins that are essential for mitochondrial homeostasis (biogenesis, apoptosis induction, Ca^2+^ signaling, transport and dynamics). By interacting with a plethora of proteins, SMN is also involved in different cell signaling pathways and in the dynamic regulation of membranes and cytoskeleton. All these SMN functions are ultimately crucial for mitochondrial biogenesis, Ca^2+^ signaling, transport and dynamics. The figure was created with BioReneder.com.

SMN is ubiquitously expressed but particularly high in the spinal cord [22,23] and has a plethora of housekeeping and cell-type specific functions, such as snRNP biogenesis, micro-RNA biogenesis, splicing, mRNA transport, translation, DNA-damage and degradation, R-loop formation, stress response, cell signaling, Ca^2+^ homeostasis, actin and microtubules dynamics, vesicle trafficking, autophagy, endocytosis and mitochondrial homeostasis (Figure 2) [13,24,25]. The SMN protein interacts directly or indirectly with >60 proteins [24,26]. This explains the dramatic effect seen in most cells, when levels drop below 10–20%, as in type 0 or I. However, when 30–40% of SMN remains, as in the case of SMA type II-IV, motor neurons are mainly affected, suggesting a very important and specific role of SMN in these cells [13]. Despite this, we still do not understand why certain motor neurons are more vulnerable than others are or why sensory neurons are not affected or are less affected than motor neurons, despite also having very long axons.

Rarely, some individuals—most often siblings of SMA patients or parents of SMA children—carry homozygous *SMN1* deletions and identical *SMN2* copies as the affected sibling(s) but do not exhibit SMA symptoms, strongly indicating protection via SMA modifying gene (s). In humans, two protective modifiers/cellular processes have been reported so far: upregulation of plastin 3 (PLS3) and downregulation of neurocalcin delta (NCALD) [27,28,29]. PLS3 is an F-acting binding and bundling protein with an essential role in endocytosis, cell migration, Ca^2+^ homeostasis, translation and others [30], while NCALD is a neuronal calcium sensor protein involved in Ca^2+^ homeostasis, endocytosis and G-protein coupled receptor signaling transduction [29,31]. Both modifiers restore impaired endocytosis induced by reduced SMN levels and ameliorate the SMA phenotype not only in humans but across species [27,29,32,33,34]. A plethora of other potential SMA modifiers have been identified by genetic screens, protein interaction studies or pathway analysis, as described in detail in the following review [35].

### Mitochondrial Function in SMA and SMA-Like Diseases

In recent years, an increasing number of studies, including clinical, structural and functional, have revealed various mitochondrial defects in motor neurons and muscles of many mammalian and non-mammalian SMA model systems. Mitochondria are crucial organelles present in eukaryotic cells that provide most of the cellular ATP supply. Mitochondrial ATP production occurs through a process called oxidative phosphorylation, mediated by the electron transport chain (ETC) complexes of the inner mitochondrial membrane. In addition to ATP production, mitochondria have many other important roles. They can store Ca^2+^ ions contributing to Ca^2+^ homeostasis and signaling, they are source of reactive oxygen species (ROS) and are the seat of many important metabolic pathways. Furthermore, mitochondria are crucial mediators of stress responses and cell death. Interestingly, in a recent study, we examined unsolved SMA-like cases by whole exome or whole genome sequencing, and in 47% of the solved cases we identified pathogenic variants in genes involved in mitochondrial dynamics (*VPS13D*, *DNM1L*), mtDNA replication and maintenance (*POLG*, *MPV17*), mitochondrial translation machinery (*C12orf65*) and respiratory chain (*NDUFS6*, *FDXR*, *ECHS1*) [36]. In other common neuromuscular disorders characterized by MN degeneration, such as amyotrophic lateral sclerosis (ALS) or Charcot-Marie-Tooth type II (axonal form), genes involved in mitochondrial function and biogenesis are often mutated [37,38,39]. These genetic associations strongly underlie and further support the essential role of mitochondria in motor neuron and muscle functionality.

**Figure 3:**
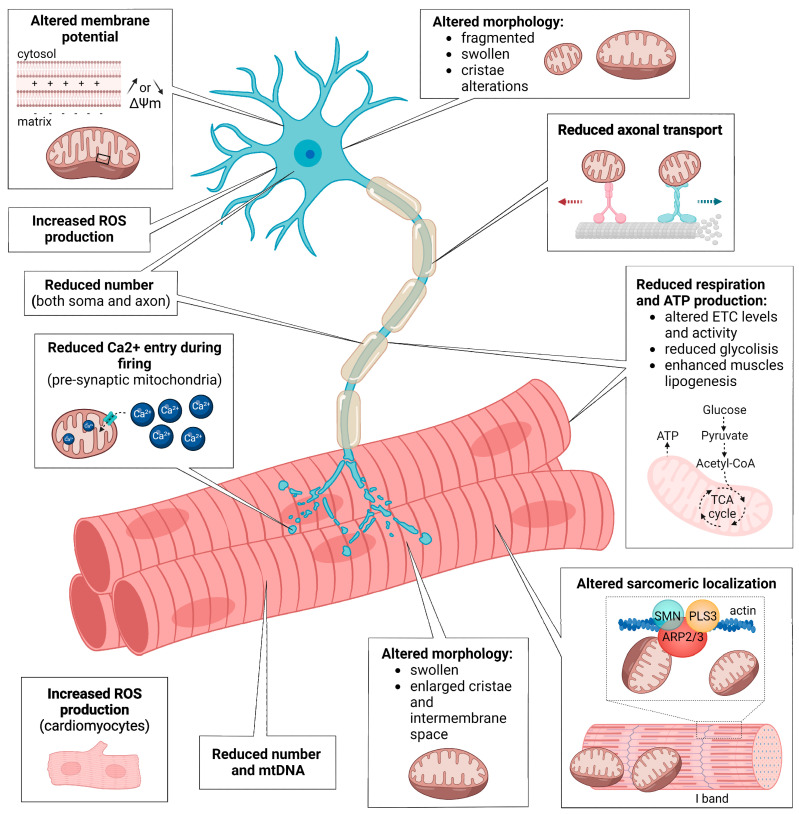
Schematic summary of the mitochondrial defects observed in SMA neurons and SMA muscles. In both MNs and muscles, SMN loss causes mitochondrial morphology aberrations, reduced mitochondria number, altered respiration, ATP and ROS production. SMN depletion affects the cytoskeleton dynamics, resulting in altered mitochondrial transport in MN axons and defective mitochondrial localization in sarcomeres. In SMA MNs, mitochondria also present defective membrane potential and, at pre-synapses, reduced Ca^2+^ influx capacity. The figure was created with BioReneder.com.

Here, we discuss the main findings, describing the different types of mitochondrial dysfunctions observed in SMA neurons and muscles (Figure 3) and how potential modifiers could ameliorate mitochondria-dependent SMA phenotypes. In the conclusion, we highlight the most burning open questions in the field and their implications for the development of potential new combinatorial therapies, especially in adults.

## 2. Mitochondria Dysfunctions in SMA Neurons

### 2.1. Role of Mitochondria in Neurons

Neurons, the primary components of the nervous system, are highly polarized non-dividing cells that require vast amounts of energy to perform and maintain their functions. The human brain accounts for about 20% of the total energetic consumption [40], therefore, neuronal cells necessitate highly functional mitochondria. In both developing and mature neurons, mitochondria provide sufficient ATP for transcription, protein translation, actin cytoskeleton remodeling, trafficking of cargoes, regulation of transmembrane ion gradients and synaptic vesicles recycling [41]. In addition to ATP synthesis, mitochondria are key players in the regulation of Ca^2+^ homeostasis, lipid biogenesis and ROS signaling [41]. As neurons are extensively polarized and subspecialized cells, newly generated mitochondria undergo anterograde active transport to ensure energy supply in all compartments, while retrograde transport moves damaged mitochondria back to the soma [42]. In developing neurons, mitochondria are concentrated at growth cones and axonal branching sites; in mature neurons, mitochondria form functional domains—upon anchoring to actin cytoskeleton and microtubules—at pre- and post-synapses [43,44,45]. This distribution of mitochondria allows local Ca^2+^ buffering and ATP production to sustain local translation, actin dynamics, and finely regulate vesicles fusion/replenishment during firing [43,44,45]. At the same time, local protein synthesis of nuclear-encoded mitochondrial genes occurs in axons, and it is crucial to maintain mitochondria morphology and membrane potential [46,47,48].

In non-dividing cells, such as neurons, mitochondrial fusion and fission balance is essential to regulate mitochondrial size, number, morphology and proper turnover via biogenesis and mitophagy. Furthermore, in neurons, mitochondrial fission is required for efficient transport of mitochondria to the axon, since the trafficking of smaller organelles is facilitated [49]. The higher level of fission in axons, compared to dendrites, explains why axonal mitochondria are typically observed as small and punctuate (up to 3 µm of length), while dendritic mitochondria are long and tubular (up to 36 µm of length) [45,50,51]. Altered mitochondrial fusion/fission dynamics has important consequences for mitochondria functionality and is implicated in many neurodegenerative disorders, including Alzheimer’s disease (AD), Parkinson’s disease (PD), Huntington’s disease (HD) and ALS [49,52].

Although SMN protein is ubiquitously expressed, the predominant SMA feature is lower alpha motor neuron loss in the spinal cord, as shown by post-mortem studies in humans that revealed decreased number, atrophy and aberrant migration of anterior horn MNs in all SMA types [53,54,55]. These defects are accompanied by neuromuscular junction (NMJ) alterations, such as immature acetylcholine receptor clusters, synaptic vesicle trafficking defects and aberrant ultrastructure of nerve terminals [56]. Transmission electron microscopy analysis of SMA animal models confirmed these structural defects. In parallel, various cellular processes, which are all altered in SMA, such as splicing and miRNA processing [57,58], mRNAs trafficking to axons terminals [59,60,61], Ca^2+^ homeostasis [62], translation [63,64], endocytosis [32,65], and cytoskeletal dynamics [66,67,68] can lead to the mitochondrial dysfunctions observed in SMA and other MN disorders.

### 2.2. Mitochondrial Morphology, Dynamics and Transport in SMA Neurons

An important indicator of mitochondria functionality is mitochondrial morphology. Transmission electron microscopy and immunofluorescence staining revealed alterations of mitochondria shape both in tissues and in cultured MNs. In spinal cords from mouse models of different SMA-types, mitochondria are mainly fragmented (smaller and more spherical). In addition, some mitochondria display reduced cristae density and/or lamellar inclusions, while others are swollen or vestigial [69,70,71]. The examination of the nerve terminals at the NMJ of murine transverse abdominal muscle and diaphragm revealed similar defects [67,72,73]. General reduction in mitochondrial size is also observed in murine and human in vitro MN models of SMA [69,74,75,76]. All together, these results suggest that mitochondria fragmentation is enhanced from early stage of development in all SMA types. Increased mitochondrial fragmentation may result from impaired fusion [77] or excessive fission [49,52], both implicated in the pathogenesis of many neurodegenerative diseases. Of note, decreased mitochondrial size and, importantly, decreased cristae density, may lead to low ATP synthesis efficiency [78]. Moreover, mitochondrial fragmentation and swelling are associated with cellular apoptosis [79,80], frequently described as one of the mechanisms leading to MN death in SMA [81,82,83,84,85].

Many studies revealing altered mitochondrial morphology showed concomitant reduction in the number and density of mitochondria, especially along MN axons [66,71,75,86,87]. A general reduction in mitochondrial number may derive from altered biogenesis and/or excessive mitophagy, while defective mitochondria distribution along MN axons might be the result of an impaired transport. Live-imaging studies in primary MNs from different murine SMA models showed reduced retrograde axonal transport of mitochondria [69]. Similar trafficking alterations are present in MNs differentiated from human pluripotent stem cells, supporting the impact of SMN reduction on mitochondrial transport [74,76]. Cytoskeletal defects may explain the MN mitochondria trafficking alterations, since neurofilament accumulation and perturbed actin and microtubules dynamics are widely described in SMA [66,67,68,72,87]. Genetic manipulation of microtubules dynamics regulators can rescue SMA mitochondrial trafficking defects. Stathmin, a phosphoprotein that promotes microtubules depolymerization, and MAP1B, a microtubule-associated protein regulating α-tubulin detyrosination, are up-regulated in murine cellular SMA models, and their depletion restores proper mitochondrial transport and localization, as well as ameliorate SMA phenotypes [66,87]. Furthermore, the down-regulation of mitochondrial motor proteins, such as KIF1B and KIF1BP, upon SMN loss can also affect mitochondrial transport, as observed in SMA murine primary MNs [75].

### 2.3. Mitochondrial Respiration and Metabolism in SMA Neurons

Altered mitochondrial morphology has severe implications on mitochondrial functionality and bioenergetics [88]. Decreased basal and ATP-linked mitochondrial respiration rate, as well as decreased proton leak, occur in both SMA murine MN cultures and in *Smn*-depleted zebrafish embryos [69,89]. As a consequence, low levels of ATP are detected in murine cellular models and spinal cord homogenates [75,89,90]. A recent study demonstrated that neurons can face energy-demanding conditions by obtaining ATP through glycolysis [91]. Nevertheless, evidence suggests that in SMA the energy-deprivation status is not compensated by increased glycolysis [75,89]. Indeed, murine primary MNs showed reduced glucose uptake [75]. A comparative gene expression profiling of murine MN pools—with different SMA vulnerability—showed that phosphoglycerate kinase 1 (PGK1), a key glycolytic enzyme, is specifically down-regulated in SMA vulnerable MNs, and, when overexpressed or pharmacologically activated, can ameliorate SMA phenotype in zebrafish embryos [89]. These observations highlight the importance of glycolysis in specific MN pools to potentially counteract SMA mitochondrial bioenergetics defects. Altered regulation of ETC complexes [70,75], particularly of complex I [75], revealed by proteomic studies in murine MNs, may also cause abnormal mitochondrial respiration. In agreement with proteomics data, dysregulations of ETC complex I and IV activities were measured in murine SMA-like cells [90] and primary MNs [75]. On the other hand, a study in MNs and astrocytes differentiated from SMA patients-derived induced pluripotent stem cells (iPSCs) did not detect changes in the protein levels of complex II and IV [92], although this does not exclude a possible alteration of their activities. Interestingly, in one study the analysis of mitochondrial respiration both in murine primary spinal MNs and forebrain neurons showed defective respiration only in the former [69]. The fact that spinal MNs are fast fatigable, with high metabolic rate and under constant energetic stress, may explain why defects in mitochondrial respiration and ATP production predominantly affect this neuronal type.

### 2.4. Mitochondrial Oxidative Stress and Ca^2+^ Homeostasis in SMA Neurons

Altered mitochondrial respiration and ATP production often results in increased mitochondrial oxidative stress in neurons [93]. Many studies in post-mortem tissues from SMA patients and cellular models showed increased ROS levels both prior and after SMA-phenotype onset. Autopsies in children affected by SMA type I showed abnormal accumulation of 4-hydroxy-2-nonenal, a reactive lipid aldehyde, resulting from lipid peroxidation in brainstem and spinal MNs [94]. This is the only evidence for a specific type of ROS implicated in SMA neurons, since all the other studies in murine and human cellular models demonstrated a general increase in total or mitochondrial ROS production via unspecific probes or dyes [69,75,85,90,92,95]. Of note, one study, besides finding no evidence of altered mitochondrial respiratory complexes in iPSCs-derived MNs and astrocytes, reported normal mRNA levels of ROS markers and antioxidant enzymes, as well as no sign of DNA peroxidation and catalase alterations in spinal cords of SMA mice [92]. Another study also reported no signs of oxidative stress in SMA murine spinal cords [96], although no mitochondrial-specific ROS marker was analyzed. In contrast, most of the reports, suggest increased cellular and mitochondrial oxidative stress in SMA MNs, and contrasting findings can be attributed to the different model systems, the large variability in iPSCs-MNs cultures, and the time of development at which the measurements are performed. As the alteration of mitochondrial respiration was observed specifically in SMA-MNs [69], an increased mitochondrial ROS production was found in human stem cells-derived MNs and not in forebrain neurons [95], thus suggesting a role of mitochondrial oxidative stress in determining spinal MN-specific degeneration in SMA.

Presynaptic mitochondria have a fundamental role in buffering cytosolic Ca^2+^ and producing ATP to sustain the synaptic vesicle cycle during neuronal firing [44]. To the best of our knowledge, synaptic mitochondria Ca^2+^ dynamics during electric activity were investigated only recently ex vivo in motor nerve terminals from SMA mice [97]. This study revealed that Ca^2+^ entry during neural firing is significantly lower in SMA presynaptic mitochondria, while free Ca^2+^ levels in the mitochondrial matrix are normally at rest. This is likely due to an intrinsic reduction in the mitochondrial Ca^2+^ influx capacity, possibly due to defective influx rate through the mitochondrial calcium uniporter (MCU). However, this hypothesis was not investigated. Low Ca^2+^ influx capacity of mitochondria during electrical activity may decrease their ability to produce ATP to sustain synaptic vesicles fusion (exocytosis) and replenishment (endocytosis), both defective in SMA [32,67,86].

### 2.5. Mitochondrial Membrane Potential and Apoptosis in SMA Neurons

Alterations in respiratory complexes and increased mitochondrial oxidative stress are often associated with impaired mitochondrial membrane potential. Indeed, studies in murine and human stem cells-derived SMA MNs showed a reduction in mitochondrial membrane potential (ΔΨm) [69,74], which could result from the observed decrease in mitochondrial respiration [69], complex I activity [75] and ATP production [69,89]. Moreover, a decreased ΔΨm triggers mitophagy via fission or PINK1-Parkin pathways [52], and could explain the observed reduced number of total and functional mitochondria in SMA MNs not only in axons [66,71,74,75,76,86] but also in cell bodies [69,70]. On the other hand, another study in a murine SMA cellular model found increased ΔΨm attributed to a concomitant raise in complex IV activity [90]. These discrepancies might be due to different systems and probes used to detect ΔΨm. For example TMRE (tetramethylrhodamine-ethylester) or TMRM (tetramethylrhodamin-methylester) probes, that revealed mitochondrial depolarization, are considered as more reliable than JC1 dye because of their higher sensitivity [98]. Future studies should clarify whether mitochondrial depolarization or hyperpolarization is predominant in SMA neuronal mitochondria, since mitochondrial membrane potential abnormalities are strongly connected to cell death. In particular, mitochondrial persistent depolarization can lead to the opening of the mitochondrial permeability transition pore (mPTP) with consequent induction of mitochondrial swelling and apoptotic factors release [99].

Mitochondria can trigger cell death through the intrinsic apoptotic pathway, via cytochrome c release and subsequent activation of caspases 3/7 [100,101]. This can occur either following mitochondrial dysfunctions or as a consequence of internal stimuli, such as DNA damage, hypoxia, Ca^2+^ overload or oxidative stress. Evidence for MNs death through apoptotic pathways in SMA was provided by many studies both in patient tissues [81] and in animal and cellular models [82,83,102,103,104]. Some findings indicate mitochondrial mediation of apoptosis in SMA. For example, altered levels of mitochondrial pro-apoptotic proteins, such as BCL2, P53 and cytochrome c, were detected in spinal cords from SMA patients and in neuronal cellular SMA models [81,103]. Additionally, SMN is a putative interactor of both BCL2, a mitochondrial anti-apoptotic protein [105,106], and P53, a protein that can induce apoptosis through several mechanisms, including mitochondrial release of pro-apoptotic factors [107]. SMN interaction with these proteins prevents apoptosis onset [105,107], thereby suggesting involvement of the intrinsic apoptotic pathway in SMA cell death. Intrinsic apoptotic pathway can also be triggered by neuronal apoptosis inhibitory protein (NAIP) depletion, which is often present in SMA, since it is encoded by a gene located in the same genomic region of *SMN1* [108]. Furthermore, recent studies on human stem cells-derived MNs showed that indirect boosting of mitochondrial function via Levetiracetam treatment results in a reduction in caspase 3/7-mediated apoptosis not accompanied by an increase in SMN levels [109]. We can conclude that the intrinsic apoptotic pathway in SMA is not merely triggered by the loss of SMN binding to mitochondrial-related apoptotic proteins, but it is also a direct consequence of mitochondrial dysfunctions.

### 2.6. Regulation of Transcription and Translation of Mitochondrial Proteins in SMA Neurons

The mentioned mitochondrial alterations and further potential dysfunctional mitochondrial pathways may be tracked back to altered levels of many nuclear-encoded mitochondrial transcripts in SMA-affected MNs (Table 1). There is also evidence of altered mitochondrial-mRNA levels specifically in somato-dendritic and axonal compartments of murine SMN-depleted MNs [110]. The direct connection between SMN protein loss and mitochondrial transcripts alteration has not yet been demonstrated. Nevertheless, given that SMN is directly involved in mRNA transcription [111], splicing [57], transport [59,60,61,63,112] and translation [63,64,75,113], it is tempting to speculate that SMN depletion may affect mitochondrial transcripts at all these different stages of their biogenesis. Importantly, transcripts encoding mitochondrial proteins are among the most abundantly enriched mRNAs in MN axons, remarking the strong impact that altered mRNA trafficking and local translation might have on mitochondria functionality. Here, collecting many transcriptome studies executed in SMA model systems and reporting all the mitochondrial transcripts that are either differentially expressed, or associated to SMN protein [114] or translated by SMN-primed ribosomes [113], we aim to give an overview that might help to shed light on the connection between SMN loss and dysfunctional mitochondria. An intriguing possibility is the identification of interesting candidates that might be transported via SMN-RNA granules or locally translated by SMN-primed ribosomes [113].

## 3. Mitochondria Dysfunctions in SMA Muscles

### 3.1. Role of Mitochondria in Muscles

Muscles are high metabolic tissues that constitute almost 50% of the human body mass. Being high-energy consumption cells, myocytes contain 2–10% mitochondria by volume [115]. Skeletal and cardiac muscle cells, similar to neuronal cells, are non-dividing cells with an exceptionally complex and organized cytosol. Myocytes contain two distinct populations of mitochondria: the subsarcolemmal mitochondria, which supply ATP for gene transcription and membrane transport, and the intermyofibrillar mitochondria, which provide ATP for muscle contraction [116]. The intermyofibrillar mitochondria resemble a “power grid”, where organelles are compacted in cylinders (up to 1 µM diameter) between myofibrils and connected through protruding mitochondrial nanotunnels (diameter 26.1–204.2 nm) [117,118]. Unlike tissues with high cellular turnover where damaged mitochondria are segregated and dispersed among newly dividing cells, myocytes can only rely on mitochondrial quality control mechanisms to maintain a functional mitochondrial network [119]. Functional fusion, fission and transport are required to not only ensure uniform distribution of energy and metabolites but also of functioning mitochondria and to facilitate the clearance of the damaged organelles through mitophagy [120]. In addition to serving as an essential energy source for muscular fibers contraction, mitochondria are essential players in muscular Ca^2+^ and ROS signaling. Ca^2+^ is released and re-uptaken from the sarcoplasmic reticulum to induce muscle contraction. Intermyofibrillar mitochondria tethered to the sarcoplasmic reticulum actively participate to this process using the Ca^2+^-influx to stimulate ATP production [121] and, thus, defects in the muscle mitochondrial network morphology and Ca^2+^ homeostasis can ultimately affect mitochondrial respiration and cause oxidative stress [122].

Although the distinctive feature of SMA is low motor neuron degeneration, the loss—or partial loss—of SMN function affects most, if not all, tissues [123], and, in particular, smooth and skeletal muscles [124,125]. CT scan and histological analysis of muscle fibers from SMA patients show different levels of generalized atrophy that can vary even among the distinct muscle types of single individuals [126,127]. The diaphragm displays the earliest defects in both SMA patients and SMA model mice [70,72,73]. The cause of muscular atrophy in SMA is to be attributed to both progressive denervation [128,129] and loss of SMN specific functions in muscle cells. Nearly complete depletion of SMN (90%) in stable murine myoblast cell lines shows decreased number of gems, also observed in chicken cells [130], and decreased proliferation—but not increased cell death—and abnormal myotubes formation in vitro, due to impaired myoblast fusion [131]. Primary myoblasts derived from different SMA mouse models showed delay in skeletal muscle development due to decreased levels of expression of myogenic genes [132,133]. A recent study demonstrated that skeletal muscle-specific depletion of *Smn* (one copy of *SMN2* still present to resemble severe SMA genotype) in mice is sufficient to recapitulate the muscular fibers abnormalities typical of SMA, and, more importantly, to cause NMJ defects, despite normal SMN expression in neuronal cells [134]. However, muscle-specific expression of SMN cannot rescue neither the phenotypes nor the survival of mice affected by SMA [135]. This is because the effects of SMN loss are synergically affected by the denervation. These observations further reinforce the systematic nature of SMA and the important role of SMN in development [136]. Given the crucial and ubiquitous role of SMN, its dysfunction causes alterations that are common across tissues and conserved from fish to mammals [137,138], albeit the cell-specific molecular mechanisms that induce muscle degeneration upon SMN deficiency are still unclear.

### 3.2. Mitochondria Morphology, Dynamics and Transport in SMA Muscles

Similar to neuronal cells, SMA muscle cells are characterized by heterogeneous mitochondrial defects. As discussed above, the overall muscle architecture is strongly impaired in SMA patients. In mice, SMA muscles displaying disarranged fibers and myofibril bundles, also present the loss of sarcomere structure along with decreased number of mitochondria [67,73]. Reduced number of functional mitochondria was also observed through histochemical and biochemical analysis of muscle specimens from SMA patients. Multiple studies reported decreased mitochondrial respiratory complex activity [139,140,141,142,143,144,145], together with decreased levels of enzymatic markers (e.g., citrate synthase, ETC complex I, II, IV) and mtDNA content [140,141,145,146]. Furthermore, the highly organized mitochondria power grid associated with the sarcoplasmic reticulum is collapsed, resulting in extremely swollen mitochondria with enlarged cristae and intermembrane space [129,147,148].

As previously mentioned, the morphology of the mitochondrial network has great impact on the homeostasis of these organelles. Proper mitochondrial morphology is maintained by the correct equilibrium between fusion and fission, and by the association of mitochondria to the cytoskeleton [149]. SMN loss affects the cytoskeleton by altering the expression of numerous factors implicated in actin and microtubule dynamics [27,66,87,150,151,152], with possible repercussions on mitochondria. Indeed, in *C. elegans*, muscles-specific knockout of *smn-1* causes mitochondrial morphology and distribution defects in the sarcomere, due to the consequent decreased activity of ARP2/3 protein complex [153]. ARP2/3 complex is an actin nucleation factor, essential for actin filament polymerization and branching and for maintaining a functional actin cytoskeleton [154]. In particular, the ARP2/3 complex has a fundamental role in mitochondrial dynamics and transport, since it promotes actin polymerization at mitochondrial fission sites [155]. Interestingly, the SMA protective modifier PLS3 directly influences the ARP2/3-mediated actin-based dynamics and, thus, may influence mitochondrial dynamics and distribution [156].

Strong evidence for SMN involvement in actin dynamics and consequent impairment of myotubes formation and sarcomere structure is present in mammalian systems. For example, in murine myoblasts, SMN loss results in defects in cell migration and focal adhesion that ultimately prevent myoblast fusion in vitro [133]. In mammalian cells, SMN has been shown to directly interact with profilin2a, an actin-binding protein that regulates actin polymerization [150,151]. In healthy human muscle fibers, SMN co-localizes with profilin2a and titin in the sarcomere, as is also previously shown in *D. melanogaster* and mice [157,158]. In hypertrophic SMA myofibers, SMN localization is altered and is accompanied by aberrant accumulations of actin filaments, suggesting that SMN plays a role in actin proteostasis and the maintenance of the sarcomere architecture [159]. However, consequent potential alterations of the mitochondrial network have never been investigated in mammalian muscles cells.

### 3.3. Mitochondria Respiration and Metabolism in SMA Muscles

Decreased ADP-dependent O_2_ consumption, indicative of defective mitochondria respiration and OXPHOS, has been measured in mitochondria isolated from murine SMA muscles and in human SMA myoblasts [160,161]. The defects in energy production may arise from altered expression and regulation of the mitochondrial ETC components and/or enzymes involved in glycolysis, TCA cycle and fatty acid metabolism [162]. SMA mice display, indeed, reduced enzymatic activity of the ETC complex I, II and IV in muscles, together with enhanced lipogenesis [160]. Similarly, reduced glycolysis and increase in accumulation of triglycerides have been observed in murine SMA liver [163], and urine samples from SMA patients are also characterized by increased short-chain or medium-chain fatty acids caused by downregulation of beta-oxidation [164,165]. The systemic dysfunctional mitochondrial fatty acid metabolism may determine the development of obesity, insulin resistance and hyperinsulinemia observed in young SMA patients and mice [166,167,168,169,170].

### 3.4. Mitochondrial Oxidative Stress and Apoptosis in SMA Muscles

The impaired activity of enzymes involved in mitochondrial metabolism and respiration can generate ROS and cause oxidative stress. The analysis of SMA mice heart showed increasing levels of vascular angiotensin II type 1 receptor (AGTR1), indicator of oxidative stress in cardiac tissue [171,172]. Although myocardial fibrosis in SMA mice has been attributed to increased oxidative stress, the role of ROS in SMA pathogenesis in skeletal muscles has been overlooked [171].

ROS are physiologically produced during muscular contraction, as a by-product of enhanced mitochondrial respiration, and maintained at homeostatic concentrations by the balanced activity of ROS-producing and ROS-scavenging enzymes [173]. Transient increase in ROS production promotes muscle fiber adaptation to both strong contractions and immobility; on the contrary, oxidative stress caused by prolonged high ROS levels can directly affect proteins, lipids and nucleic acids, as well as trigger cell death signaling cascades [174,175]. Furthermore, it has been recently shown that SMN overexpression can reduce cytochrome c release and caspase-3 activation (mitochondrial apoptotic pathway [176]) in murine cardiomyocytes, by increasing the expression of BCL2 and decreasing the expression of BAX, anti- and pro-apoptotic factor, respectively [177]. In addition, chemically induced ROS production causes aberrant splicing of *SMN2* in SMA mice in heart and skeletal muscle, resulting in even lower level of functional SMN protein [178]. Therefore, oxidative stress caused by mitochondrial dysfunctions and the consequent even lower levels of SMN protein could synergically enhance cell death rate and, thereby, promote muscular atrophy and cardiovascular abnormalities in SMA patients [179].

### 3.5. Regulation of Transcription and Translation of Mitochondrial Proteins in SMA Muscles

Since SMA is widely recognized as a motor neuron pathology, most of the studies investigating the cellular phenotypes, including mitochondrial functions, arising from SMN depletion are carried out in neural cell models [180]. Although muscular aberrations are often considered secondary lesions derived from loss of neuromuscular junction and denervation, we discussed in this review how SMN ablation is sufficient to cause cell-autonomous defects also in myocytes [131,134]. Mitochondria play a major role in muscle function and development, and, given that muscular phenotypes of SMA resemble many different mitochondriopathies [181,182], it is likely that mitochondrial dysfunctions also play a major role in SMA pathogenesis. Although there is currently no evidence of specific mitochondrial functions of SMN in myocytes, the expression of many essential mitochondrial proteins may be affected by SMN loss, as suggested from the transcriptome datasets summarized in Table 1. Further investigations in this direction could identify new modifying proteins/pathways that rescue or mitigate the mitochondrial defects caused by SMN deficiency in muscles.

Moderate exercise represents an accessible and effective therapeutic approach to stimulate and improve mitochondria functionality in muscle fibers [183]. SMA patients benefit of exercise therapy to improve muscular strength and size [184,185,186,187]. In SMA mice, mitochondrial function analysis showed that individuals subject to regular exercise display activation of the AMPK–p38–PGC-1*α* signaling axis and higher O_2_ consumption in skeletal muscles, ultimately resulting in decreased lipogenesis [160,188]. Similarly, chemical stimulation of the AMPK-p38 pathway using celecoxib, a cyclo-oxygenase 2 inhibitor, increases the lifespan and the motor function of SMA mice [189]. These observations suggest that targeting muscle-specific mitochondrial abnormalities could alleviate not only muscular weakness but also secondary systemic disorders, such as hyperglycemia, hyperglucagonemia, glucose resistance and dyslipidemia, observed in SMA patients [190,191]. Future studies must focus on the function of SMN on mitochondria homeostasis in muscle cell models to elucidate the molecular mechanisms that contribute to SMA etiology and to identify new potential modifiers and therapeutic targets.

## 4. Mitochondrial Functions: Potential SMA Modifiers?

The studies discussed here clearly demonstrate that mitochondrial dysfunctions are widespread in SMA tissues across species. Although the studies performed on motor neurons outnumber the studies in muscles or other cell types, SMA deserves to be considered and studied as multi-systemic disorder. Mitochondria dysfunctions arise from the early stages of SMA pathogenesis and may play a crucial role in the disease progression, even by impacting directly on SMN protein level. For example, increased mitochondrial ROS production can directly affect *SMN2* splicing, further reducing functional SMN levels [178]. Additionally, mitochondrial mitofusin 2 (MFN2) mediate the transport of calpastatin along MN axons, locally inhibiting calpain protease activity and preventing NMJ degeneration [192]. As SMN degradation is also performed by calpains, altered mitochondrial transport might affect SMN turnover in axonal terminals [76].

Given the diversified functions of SMN protein, it is not surprising that mitochondria are downstream targets (Figure 2). Dysfunctional RNA metabolism affects a plethora of mitochondrial-related transcripts encoding for proteins involved in all mitochondrial functions (Table 1). Additionally, altered ubiquitination, due to defective UBA1 splicing, impacts on Wnt/β-catenin signaling, a strong activator of mitochondria biogenesis [193,194]. Furthermore, as previously discussed, cytoskeletal alterations linked to SMN deficiency strongly affect mitochondria transport, dynamics and morphology. Interestingly, many of the SMA modifiers described so far act on the above-mentioned pathways, resulting, in some cases, in complete reverse of the symptoms (e.g., PLS3 and NCALD). Thus, we speculate that boosting mitochondrial activity could lead to a partial rescue of SMA degenerative phenotypes. For instance, we already discussed how enhancing the activity of proteins involved in cytoskeletal dynamics, such as PLS3, stathmin, MAP1B and additionally CORO1C [195], have positive effects on both mitochondrial functionality and SMA phenotypes. In a recent study on *Smn*-depleted zebrafish, overexpression of necdin, a protein promoting mitochondrial biogenesis in neurons, could rescue axonal outgrowth defects [89]. Another known SMA modifier that may exert its protective function acting on mitochondria is stasimon, a suppressor of p38-MAPK cell death pathway that is decreased in SMA due to splicing defects [196]. A recent study shows that stasimon localizes in correspondence of ER-mitochondria contact sites, possibly participating to mitochondria quality control, dynamics and calcium-handling mechanisms [197]. Lastly, many other described SMA-modifiers, such as PTEN [84], ZPR-1 [198] and NAIP [108], reduce mitochondrial-induced apoptosis. These findings suggest that modulating the expression of modifier genes involved in mitochondrial functions may be an effective strategy to mitigate SMA phenotypes. Given the strong importance of mitochondria in all cell types, and especially in MNs and muscles, we are convinced that future studies should focus on the identification of new mitochondrial-related modifiers of SMA pathology. Genetic screenings for new modifiers recently conducted both in *D. melanogaster* [199] and human HEK cells [200] highlighted new potential mitochondrial candidates (Table 1).

Currently, there is only one pharmacological therapy specifically targeting mitochondria that reached the clinical trial stage for SMA patients: olexisome. Olexisome modulates the mitochondrial permeability transition pore preventing cell death induction [201]. Unfortunately, olexisome administration in phase II trial failed due to ineffective long-term results [202]. Nevertheless, given the promising effects of this compound in several in vitro and in vivo models of motor neuron disorders—including a severe mouse model of SMA—the possibility of using it in combinatorial therapies should not be neglected [201]. Nowadays, the research in the field of mitochondrial-targeted drugs is moving forward and an increasing number of new molecules will be available to be tested in many different SMA models [203]. Recently, in vitro studies on iPSCs-derived MNs showed efficacy of several compounds (some of them already used to treat ALS or epilepsy) in rescuing both mitochondrial dysfunctions and SMA phenotypes—with or without increasing SMN protein levels—adding new promising candidates for a therapy [74,76,85,109].

## 5. Conclusions

As key bioenergetics organelles, mitochondria may play a major role in SMA pathogenesis. The activity of SMN protein has an impact on many cellular processes and mitochondrial alterations are among the downstream effects of SMN loss (Figure 2). In SMA pathological conditions, specific mitochondrial abnormalities may affect other related mitochondrial functions, thereby amplifying SMN-dependent cellular alterations. Still, the causal link between SMN loss and mitochondrial dysfunctions is unknown. Future cellular studies must focus on the ambitious goal of unravelling the molecular mechanisms that directly connect SMN activity with mitochondrial homeostasis. We speculate that delving into the altered mitochondria-related transcripts, observed in various SMA models (Table 1), is key to advance our knowledge in this direction. Many known mutations in genes encoding mitochondrial proteins result in SMA-like disorders, further remarking the crucial role of mitochondria in the development of SMA phenotypes and symptoms. In fact, mitochondrial pathways represent a valuable target not to cure SMA, but rather to mitigate the symptoms, improve the life quality of patients and possibly slow down the development of the disease.

**Figure 4:**
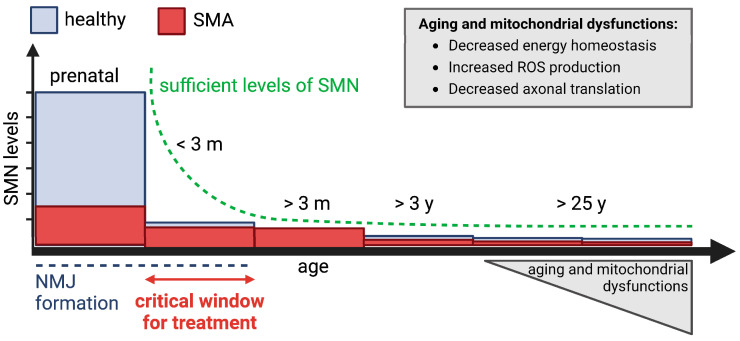
Cellular aging and mitochondrial aberrations may decrease MN performance below their functional threshold in adult SMA. SMN protein functions are mainly required during the development; indeed, also in healthy individuals the levels of SMN protein drop already after birth by 6.5-fold. SMN is also required in the adulthood but only at low levels, not significantly different between healthy and SMA individuals [12]. Many aging factors, such as decreased bioenergetics, increased ROS production and decreased axonal translation, may drop the function of NMJs just slightly below the threshold, ultimately causing SMA in adults. The figure was created with BioReneder.com.

Many aspects of SMA etiology, however, are still poorly understood. Intriguingly, although in adulthood very low SMN levels are sufficient to maintain the health of MNs [12], there are patients carrying 4–6 *SMN2* copies that develop SMA in adulthood, sometimes even after 30 years of age [7,204]. We hypothesize that in such cases, cellular aging—a natural phenomenon associated with increased ROS production, decreased mitochondrial bioenergetics and protein translation—affects the MNs (already vulnerable due to reduced SMN levels during development), dropping below a functional threshold that is compatible with health, and thereby triggering SMA onset (Figure 4). We speculate that the “watch and wait” strategy, adopted for SMA individuals with four or more *SMN2* copies, may overshoot the most critical period when high SMN levels are required for NMJ development and maturation. Delaying the treatment, instead of starting it in the first post-natal months up to three years, may affect the efficacy of the therapy. Indeed, after 3 years of age, minimal SMN levels are required and there is no significant difference between SMA and healthy individuals [12]. Even more, it has been shown in mice that inducing constantly elevated SMN expression (scAAV9-SMN gene therapy) in MNs, above the endogenous levels, is neurotoxic in adult animals [205]. Therefore, even in newborns with four *SMN2* copies, early treatment and supply of sufficient SMN protein during the critical time window of NMJ maturation may be key to support their best possible development and to confer resistance against deleterious aging effects later in life. For this purpose, SMA newborn screening is a determinant to identify SMA individuals pre-symptomatically and promptly start the treatment. Lastly, monitoring new therapeutic advances, especially when targeting mitochondrial processes, for the treatment of other late-onset MN disorders, such as ALS, might be extremely helpful. Indeed, we speculate that adult SMA patients may rather benefit from therapies ameliorating mitochondrial functions than therapies elevating SMN levels.

**Table 1:** mRNA of genes encoding mitochondria-associated proteins that are either altered upon SMN loss, associated to SMN, translated by SMN-primed ribosomes or potential SMA modifiers.

Mitochondrial Function	Accession Number	Gene Symbol	Tissue/Cell Type	Organism	Refs.
Altered mRNA Levels					
ROS signaling and oxidative stress	NM_001302272	*PRDX3*	iPSCs-motor neurons	human	[104]
	NM_001286264	*MRS2*			
	NM_000305	*PON2*	primary muscle cultures	human	[206]
	MGI:104887	*Gpx1*	spinal cord	mouse	[207]
	MGI:96916	*Maob*			[208]
	MGI:1921607	*Efhd1*			
	MGI:104767	*Gpx4*			
	MGI:1916617	*Glrx2*	spinal motor neurons		[69]
	NM_001198532	*OXR1*	iPSCs-motor neurons, motor neurons (axonal compartment)	human, mouse	[104,110]
OXPHOS	NM_001001935	*ATP5A1*	iPSCs-motor neurons	human	[104]
	NM_001202469	*GBAS*			
	NM_001686	*ATP5B*			
	NM_001042546	*ATPAF1*			
	NM_003366	*UQCRC2*			
	NM_001282419	*NDUFA5*			
	NM_006886	*ATP5E*			
	NM_001319036	*COX7A2L*			
	NM_001008215	*C2orf64*			
	NM_002489	*NDUFA4*			
	NM_001099668	*HIGD1A*			
	NM_001002258	*ATP5G3*			
	NM_004374	*COX6C*			
	NM_001001973	*ATP5C1*			
	NM_001865	*COX7A2*			
	NM_001867	*COX7C*			
	NM_001864	*COX7A1*	primary muscle cultures		[206]
	NM_004146	*NDUFB7*	muscle biopsy		[127]
	MGI:192462	*C5orf63*	spinal cord	mouse	[208]
	MGI:1349919	*Ndufb11*	motor neurons (somatodendritic compartment)		[110]
	MGI:106362	*Sco1*	motor neurons (axonal compartment)		
	MGI:1922656	*Ndufs7*	spinal motor neurons		[69]
	MGI:107801	*Atp5b*			
	MGI:1343103	*Ndufa2*			
	MGI:106100	*Etfdh*			
	Dr.113730	*latro*	embryo	zebrafish	[209]
	T06D8.5	*cox-15*	larvae	worm	[210]
	NM_001916	*CYC1*	muscle biopsy, spinal motor neurons	human, mouse	[69,127]
Fatty acid metabolism	NM_000182	*HADHA*	iPSCs-motor neurons	human	[104]
	NM_001007098	*SCP2*			
	NM_016243	*CYB5R1*	primary muscle cultures		[206]
	MGI:1859310	*Asah2*	spinal cord	mouse	[208]
	MGI:894291	*Acsl6*	motor neurons (somatodendritic compartment)		[110]
	MGI:1928939	*Acot9*	motor neurons (axonal compartment)		
	MGI:1196345	*Agpat5*			
	MGI:1914702	*Pnpla8*			
	MGI:109501	*Crat*	spinal motor neurons		[69]
	CBG24278	*acaa-2*	larvae	worm	[210]
	Y48G9A.10	*cpt-3*			
	F44C4.5	*ppt-1*			
	Y45F3A.3a	*acdh-11*			
	E04F6.3	*maoc-1*			
	NM_001318509	*ACSL4*	iPSCs-motor neurons, motor neurons (axonal compartment)	human, mouse	[104,110]
Ca^2+^ homeostasis and signaling	MGI:88255	*Anxa6*	spinal cord	mouse	[207]
	MGI:1338917	*S100a1*			[208]
	MGI:1914065	*Mcub*	motor neurons (axonal compartment)		[110]
	MGI:109326	*Bnip3*	spinal motor neurons		[69]
	MGI:1278343	*Nipsnap2*			
	ZK816.5	*dhs-26*	larvae	worm	[210]
Apoptosis	NM_001191	*BCL2L1*	iPSCs-motor neurons	human	[104]
	NM_001199839	*BCL2L2*			
	NM_001270729	*BCL2L13*			
	NM_001029839	*C3orf23*			
	NM_001008388	*CISD2*			
	NM_007523	*Nbak1*	spinal cord	mouse	[207]
	MGI:1933786	*Dnaja3*			
	MGI:1346325	*Gadd45g*			
	MGI:1197519	*Bcl2l11*	spinal motor neurons		[69]
	MGI:1913744	*Prelid1*			
	MGI:99702	*Bax*			
	F23B12.9	*egl-1*	larvae	worm	[210]
	NM_001196	*BID*	iPSCs-motor neurons, spinal cord	human, mouse	[104,207]
mtDNA maintenance, mitochondrial transcription and translation	NM_133259	*LRPPRC*	iPSCs-motor neurons	human	[104]
	NM_001182520	*MRPL3*			
	NM_001243251	*NARS2*			
	MGI:191904	*Mrps27*	spinal cord	mouse	[208]
	MGI:1922869	*Fastkd2*	motor neurons (axonal compartment)		[110]
	MGI:2142973	*Lars2*			
	MGI:1917297	*Trnt1*			
	MGI:1889295	*Eral1*			
	MGI:1913660	*Mterf3*			
	MGI:2387629	*Tardbp*			
	MGI:1915541	*Mto1*			
	MGI:1351639	*Mrpl15*			
	MGI:1923776	*Gatc*			
	MGI:1920040	*Ssbp1*			
	MGI:1923686	*Tufm*	spinal motor neurons		[69]
	MGI:2137215	*Mrpl11*			
	MGI:1919049	*Ptcd1*			
	MGI:1929864	*Myg1*			
	MGI:107252	*Nsun2*			
	MGI:1333820	*Mrpl30*			
	MGI:107329	*Mrpl50*			
	MGI:107810	*Tfam*			
	W09D10.3	*mrpl-12*	larvae	worm	[210]
	Y119D3B.16	*mrpl-16*			
	C05D11.10	*mrps-17*			
	F29C12.4	*gfm-1*			
	Y46H3A.7a	*mrpl-39*			
	CBG13134a	*mrpl-14*			
	MGI:1919214	*Atad3a*	motor neurons (axonal compartment), larvae	mouse, worm	[110,210]
	NM_016622	*MRPL35*	iPSCs-motor neurons, spinal motor neurons	human, mouse	[104]
Mitochondria quality control	MGI:2135611	*Immp2l*	spinal cord	mouse	[208]
	MGI:1346017	*Clpx*	spinal motor neurons		[69]
	MGI:1926884	*Huwe1*	motor neurons (somatodendritic compartment)		[110]
	MGI:1920209	*Lonrf2*	motor neurons (axonal compartment)		
	MGI:1891828	*Becn1*			
	MGI:1915207	*Marchf5*			
	F59H6.11	*bath-5*	larvae	worm	[210]
	C37H5.8	*hsp-6*			
	Y47G6A.10	*spg-7*			
Mitochondrial dynamics, membrane trafficking	NM_001206651	*SH3GLB1*	iPSCs-motor neurons	human	[104]
	NM_001033566	*RHOT1*			
	NM_001256763	*FAM49B*			
	NM_001303249	*SLC25A46*			[104]
	NM_001164730	*REEP1*			
	NM_014394	*GHITM*			
	NM_213720	*C22orf16*	muscle biopsy		[127]
	NM_017812	*CHCHD3*			
	MGI:1313276	*Vamp1*	spinal cord	mouse	[208]
	MGI:1098586	*Rab11fip5*	motor neurons (axonal compartment)		[110]
	MGI:1918953	*Armcx3*	motor neurons (axonal compartment)		
	MGI:1914977	*Stx17*	motor neurons (somatodendritic compartment)		
	MGI:2385189	*Rap1gds1*	spinal motor neurons		[69]
	F21C10.10	*CELE_F21C10.10*	larvae	worm	[210]
	Y37E3.9	*phb-1*			
	NM_006373	*VAT1*	iPSCs-motor neurons, spinal motor neurons	human, mouse	[69,104]
Mitochondrial import, transport, translocation (metabolites, proteins, lipids, ions)	NM_014820	*TOMM70A*	iPSCs-motor neurons	human	[104]
	NM_001104647	*SLC25A36*			
	NM_001321967	*ATAD1*			
	NM_001322977	*SFXN1*			
	NM_002635	*SLC25A3*			
	NM_001270679	*CCDC90B*			
	NM_001135694	*VDAC3*			
	NM_006335	*TIMM17A*			
	NM_014765	*TOMM20*			
	NM_001184783	*VDAC2*			
	NM_001151	*SLC25A4*	muscle biopsy		[127]
	U94592	*UCP2*	primary muscle cultures		
	MGI:1343262	*Timm44*	motor neurons (axonal compartment)		[110]
	MGI:1917560	*Plscr3*			
	MGI:1917728	*Mipep*			
	MGI:1340062	*Sgk1*	spinal motor neurons		[69]
	MGI:1349215	*Abcd1*			
	R07E3.4	*CELE_R07E3.4*	larvae	worm	[210]
	C34C12.8	*C34C12.8*			
	Y71G12B.24a	*mppa-1*			
	CBG01742	*timm-17b.1*			
	B0432.4	*misc-1*			
	F55C5.5	*tsfm-1*			
	F56D1.3	*mrps-16*			
	Dr.77108	*ucp4*	embryo	zebrafish	[209]
	NM_152407	*GRPEL2*	iPSCs-motor neurons, motor neurons (axonal compartment)	human, mouse	[104,110]
Metabolic enzymes	NM_001242767	*MTHFD1L*	iPSCs-motor neurons	human	[104]
	NM_000663	*ABAT*			
	NM_001286220	*GOT2*			
	NM_001183948	*ODC1*			
	NM_001077180	*METTL9*			
	NM_001282621	*PGRMC1*			
	NM_001174097	*LDHB*			
	NM_001318900	*GLUD1*			
	NM_001282403	*MDH2*			
	NM_001482	*GATM*			
	NM_018464	*C10orf70*	muscle biopsy	human	[127]
	NM_002168	*IDH2*			
	MGI:87990	*Alas2*	spinal cord	mouse	[208]
	MGI:2661364	*Neu4*			
	MGI:1915871	*Mthfd2l*	motor neurons (somatodendritic, axonal compartment)		[110]
	MGI:2385311	*Dlat*	motor neurons (axonal compartment)		
	MGI:1346064	*Eci2*			
	MGI:1916296	*Isca1*			
	MGI:1889278	*Pdss1*			
	MGI:1099463	*Idh3g*			
	MGI:1918732	*Rdh13*			
	MGI:88590	*Cyp1b1*	motor neurons (somatodendritic compartment)		
	MGI:1306824	*Suclg2*			
	MGI:98731	*Tgm2*			
	MGI:2159605	*Acot2*	spinal motor neurons		[69]
	MGI:97770	*Prodh*			
	MGI:87867	*Acadm*			
	MGI:2180203	*Tmlhe*			
	T22B7.7	*CELE_T22B7.7*	larvae	worm	[210]
	C04E6.7	*CELE_C04E6.7*			
	T05G5.6	*ech-6*			
	F32D8.12b	*CELE_F32D8.12*			
	F57F4.1	*CELE_F57F4.1*			
	T07D3.9b	*CELE_T07D3.9*			
	R12C12.1b	*gldc-1*			
	F54D5.12	*CELE_F54D5.12*			
	F25B4.1	*gcst-1*			
	ZK669.4	*dbt-1*			
	C50F7.4	*sucg-1*			
	F46G10.7a	*sir-2.2*			
	T02G5.8	*kat-1*			
	F09F7.4b	*hach-1*			
	Y38F1A.6	*hphd-1*			
	T20H4.5	*EC:7.1.1.2*			
	F23B12.5	*dlat-1*			
	ZK652.9	*coq-5*			
Mitochondrial regulation and signaling	NM_001001924	*MTUS1*	iPSCs-motor neurons	human	[104]
	NM_001244974	*PPP1CC*			
	NM_001017963	*PTGES3*			
	NM_001318067	*MAPK10*			
	NM_001005	*RPS3*			
	MGI:1929628	*Rsad2*	motor neurons (somatodendritic, axonal compartment)	mouse	[110]
	MGI:1101055	*Ifit3*	motor neurons (somatodendritic compartment)		
	MGI:1918836	*Ifih1*			
	MGI:2446107	*Pde2a*			
	MGI:1344391	*Sh3bp5*	motor neurons (axonal compartment)		
	MGI:2159437	*Agtpbp1*			
	MGI:1927243	*Rala*			
	MGI:1919792	*Pgam5*			
	MGI:1913842	*Stoml2*	spinal motor neurons		[69]
	MGI:1915864	*Letmd1*			
	MGI:2441680	*Tmem8b*			
	C43E11.4	*tufm-1/2*	larvae	worm	[210]
	T24H7.1	*phb-2*			
	C16C10.11	*har-1*			
Altered mRNA splicing					
Mitochondrial import, transport, translocation (metabolites, proteins, lipids, ions)	MGI:88025	*Ank2*	motor neuron, neuroblastoma cells	mouse	[211]
Mitochondrial regulation and signaling	MGI:98397	*Src*	neuroblastoma cells		
mRNA associated to SMN					
Ca^2+^ homeostasis and signaling	MGI:109326	*Bnip3*	NSC-34 cells	mouse	[114]
OXPHOS	MGI:1333806	*Cox17*			
	MGI:1855697	*Atp5e*			
	MGI:99927	*mt-Atp6*			
	MGI:1930666	*Higd1a*			
	MGI:1316714	*Cox7a1*			
Mitochondrial import, transport, translocation (metabolites, proteins, lipids, ions)	MGI:1353433	*Timm8a1*			
	MGI:1347061	*Abcg2*			
Metabolic enzymes	MGI:1916884	*Clybl*			
	MGI:1098623	*Acaa2*			
Mitochondria quality control	MGI:1921392	*Lonp1*			
mtDNA maintenance, mitochondrial transcription and translation	MGI:1926237	*Mrps30*			
	MGI:2442510	*Dars2*			
	MGI:2135755	*Cox4i2*			
mRNA enriched for SMN-primed ribosomes					
OXPHOS	MGI:2143558	*Chchd10*	brain	mouse	
	MGI:104614	*Cox6c*			
	MGI:1316715	*Cox7a2*			
	MGI:1914862	*Mettl9*			
	MGI:1914514	*Ndufb8*			
	MGI:2385112	*Ndufs2*			
	MGI:1915903	*Samm50*			
	MGI:1914175	*Sdhd*			
	MGI:107876	*Uqcrc1*			
	MGI:1917794	*Tmem242*			
Fatty acid metabolism	MGI:894291	*Acsl6*			
	MGI:1915988	*Acss1*			
	MGI:1351861	*D10Jhu81e*			
Apoptosis	MGI:1339639	*Ogt*			
mtDNA maintenance, mitochondrial transcription and translation	MGI:2137221	*Mrpl20*			
	MGI:2153111	*Mrps6*			
	MGI:2443470	*Mrm1*			
	MGI:1914216	*Trit1*			
Mitochondria quality control	MGI:1916193	*Pink1*			
	MGI:98889	*Ubc*			
	MGI:2444207	*Vps13c*			
Mitochondrial dynamics, membrane trafficking	MGI:88025	*Ank2*			
	MGI:1261831	*Hap1*			
	MGI:108426	*Kif1b*			
	MGI:1349450	*Vat1*			
	MGI:1913687	*Fis1*			
	MGI:1928394	*Mtor*			
	MGI:1921393	*Opa1*			
	MGI:1925498	*Armcx1*			
Mitochondrial import, transport, translocation (metabolites, proteins, lipids, ions)	MGI:1915517	*Slc25a22*			
	MGI:1100517	*Clpb*			
	MGI:2444911	*Slc25a29*			
	MGI:2137681	*Sfxn5*			
Metabolic enzymes	MGI:1919289	*Mccc1*			
	MGI:2685870	*Pdp1*			
	MGI:87989	*Alas1*			
	MGI:88407	*Ckb*			
	MGI:1913637	*Nudt8*			
	MGI:1918993	*Coasy*			
	MGI:2385920	*Dip2a*			
	MGI:1858208	*Ech1*			
	MGI:2441982	*Aldh5a1*			
Mitochondrial regulation and signaling	MGI:97592	*Prkaca*			
	MGI:97138	*Mpv17*			
Genes for mitochondrial proteins that modify SMN levels/activity					
OXPHOS	NM_001286264	*MRS2*	HEK293 cells	human	[200]
Apoptosis	NM_003334	*UBA1*			
	NM_001291921	*RXRA*	larvae, NMJ between muscles 6/7 in A2 segment	*D. melanogaster*	[199]
Mitochondrial dynamics, membrane trafficking	NM_004958	*RPS6KB1*	HEK293 cells	human	[200]
Mitochondrial import, transport, translocation (metabolites, proteins, lipids, ions)	NM_003356	*UCP3*			
Metabolic enzymes	NM_001098	*ACO2*			
Mitochondrial regulation and signaling	NM_001144012	*TXNDC14*			

## Data Availability

Not applicable.
